# Dynamic Arterial Elastance as a Ventriculo-Arterial Coupling Index: An Experimental Animal Study

**DOI:** 10.3389/fphys.2020.00284

**Published:** 2020-04-06

**Authors:** Manuel Ignacio Monge García, Zhongping Jian, Feras Hatib, Jos J. Settels, Maurizio Cecconi, Michael R. Pinsky

**Affiliations:** ^1^Unidad de Cuidados Intensivos, Hospital Universitario SAS de Jerez, Jerez de la Frontera, Spain; ^2^Edwards Lifesciences, Irvine, CA, United States; ^3^Department Anaesthesia and Intensive Care Units, Humanitas Research Hospital, Humanitas University, Milan, Italy; ^4^Department of Critical Care Medicine, University of Pittsburgh School of Medicine, Pittsburgh, PA, United States

**Keywords:** ventricular-arterial coupling, left ventricular efficiency, dynamic arterial elastance, arterial load, effective arterial elastance, ventricular elastance

## Abstract

Dynamic arterial elastance (Ea_dyn_), the ratio between arterial pulse pressure and stroke volume changes during respiration, has been postulated as an index of the coupling between the left ventricle (LV) and the arterial system. We aimed to confirm this hypothesis using the gold-standard for defining LV contractility, afterload, and evaluating ventricular-arterial (VA) coupling and LV efficiency during different loading and contractile experimental conditions. Twelve Yorkshire healthy female pigs submitted to three consecutive stages with two opposite interventions each: changes in afterload (phenylephrine/nitroprusside), preload (bleeding/fluid bolus), and contractility (esmolol/dobutamine). LV pressure-volume data was obtained with a conductance catheter, and arterial pressures were measured via a fluid-filled catheter in the proximal aorta and the radial artery. End-systolic elastance (Ees), a load-independent index of myocardial contractility, was calculated during an inferior vena cava occlusion. Effective arterial elastance (Ea, an index of LV afterload) was calculated as LV end-systolic pressure/stroke volume. VA coupling was defined as the ratio Ea/Ees. LV efficiency (LV_eff_) was defined as the ratio between stroke work and the LV pressure-volume area. Ea_dyn_ was calculated as the ratio between the aortic pulse pressure variation (PPV) and conductance-derived stroke volume variation (SVV). A linear mixed model was used for evaluating the relationship between Ees, Ea, VA coupling, LV_eff_ with Ea_dyn_. Ea_dyn_ was inversely related to VA coupling and directly to LV_eff_. The higher the Ea_dyn_, the higher the LV_eff_ and the lower the VA coupling. Thus, Ea_dyn_, an easily measured parameter at the bedside, may be of clinical relevance for hemodynamic assessment of the unstable patient.

## Introduction

Dynamic arterial elastance, the ratio between PPV and SVV, defines the relationship between the changes in the arterial pulse pressure and SV during a respiratory cycle (Pinsky, 2006). This index has been demonstrated to predict the arterial pressure changes associated to fluid administration in hypotensive preload-dependent patients, as well as predicting the arterial pressure response to decreasing norepinephrine dose (Monge Garcia et al., 2014; Guinot et al., 2015), and is part of some hemodynamic optimization protocols for guiding fluid and vasopressor therapy (Guinot et al., 2017; Davies et al., 2019; Guarracino et al., 2019; Maheshwari et al., 2019).

The term elastance suggests that Ea_dyn_ is related to arterial stiffness. Indeed, Ea_dyn_ was initially proposed as an index of arterial tone (Pinsky, 2006). However, as Ea_dyn_ relates relative changes in arterial pressure and SV, Ea_dyn_ is unitless and should only be considered as an indirect measure of arterial stiffness or LV afterload. As usually measured, Ea_dyn_ is defined by the dynamic arterial pressure-stroke volume relationship during respiration, as both vary (Monge Garcia et al., 2014). Moreover, when the arterial tone was directly modified, as during the use of vasoactive drugs such as phenylephrine or sodium nitroprusside, Ea_dyn_ changed in the opposite way to arterial vasomotor tone (Monge Garcia et al., 2017a; Wodack et al., 2019). Besides, other factors not related to the arterial system have been involved in the subsequent Ea_dyn_ value, for example, heart rate and blood flow acceleration have been associated with changes in Ea_dyn_ (Monge Garcia et al., 2017a). How these arterial and cardiac factors affect to PPV and SVV would define Ea_dyn_. These potentially contradictory findings led to propose that Ea_dyn_ may represent an index of the coupling between the heart and the systemic circulation (Pinsky, 2006; Monge Garcia et al., 2017a). However, the hypothesis that Ea_dyn_ is related to VA coupling or whether Ea_dyn_ would track changes in VA coupling have not been studied. Thus, we analyzed the impact of various maneuvers known to affect vasomotor tone, contractility and volume status on both VA coupling and Ea_dyn_.

## Materials and Methods

The study was approved by the Institutional Animal Care and Use Committee (IACUC) at the Edwards Research Center, and all procedures were performed in accordance with the USDA Animal Welfare Act regulations (AWArs), and the Guide for the Care and Use of Laboratory Animals (ILAR, NAP, Washington, DC, United States, 2010, 8th edition). The ARRIVE guidelines were used for the elaboration of this manuscript (NC3Rs Reporting Guidelines Working Group, 2010).

### Anesthesia and Surgical Preparation

Experiments were performed in 12 female Yorkshire crossbreed pigs of 5.5 ± 0.8 (mean ± SD) months old weighting 81 ± 6 kg. Animals were maintained in a temperature and humidity-controlled rooms with a typical light-dark cycle and given standard chow and tap water *ad libitum*. Before anesthesia induction, a general physical examination was performed. If found to be stable, the animal was premedicated with an intramuscular injection of telazol (4.4 mg/kg), ketamine (2.2 mg/kg), and xylazine (1.1 mg/kg). They were orally intubated, and their lungs mechanically ventilated in a volume-controlled mode (fraction of inspired oxygen of 0.6–0.8, tidal volume of 10 ml/kg plus 100 ml compensation for dead space, respiratory rate of 15 breaths/min). Ventilatory settings were maintained unchanged throughout the experiment. Following endotracheal intubation, general anesthesia was maintained with isoflurane 1.5–2.5% and a mixture of oxygen (two animals), oxygen/air (three animals) and oxygen/air/nitrous oxide (seven animals). The level of anesthesia was adjusted and kept at the lowest possible dose. No neuromuscular blocking drugs were used. Animals received a loading dose of heparin (300 I.U./kg) with additional boluses during follow-up (mean total dose: 1040 I.U./kg) for preventing coagulation. Fluid maintenance was provided intravenously by a continuous infusion of Ringer’s lactate solution at 4 ml.kg^–1^.h^–1^. Rectal temperature was monitored and kept at 37°C (mean measured value 36.6°C) using a heating pad. Arterial blood gases were monitored periodically and acid-base balance was corrected by infusing bolus sodium bicarbonate or by changing ventilator frequency if required. The depth of anesthesia was assessed throughout the study for muscular reflex by checking the jaw tone and the toe pinch assessment. Animal anesthesia was monitored and recorded approximately every 15 min for the duration of the experimentation. All the experimental procedures were performed by the same skilled operator.

Instantaneous LV PV measurements were obtained from a 7Fr-lumen dual-field conductance catheter introduced through the left external carotid artery with 12 equidistant electrodes and a high-fidelity pressure sensor (CA71083-PL, CD Leycom, Zoetermeer, Netherlands) connected to a PV signal processor (Inca^®^, CD Leycom, Zoetermeer, Netherlands). The catheter tip was positioned in the long axis of the LV, and the correct placement was confirmed by fluoroscopy and the examination of the individual segmental LV PV loops.

### Data Collection and Analysis

The volume signal calibration was performed via right-heart catheterization with a pulmonary artery catheter (Swan-Ganz CCOmbo 777HF8, Edwards Lifesciences, Irvine, CA, United States) inserted percutaneously through the right internal jugular vein to the pulmonary artery using the standard thermodilution method for the determination of CO. The average value of 3 to 5 thermodilution boluses randomly injected during the respiratory cycle was used for volume calibration. Correction for parallel conductance (the conductance of the surrounding tissues, which was subtracted from the raw catheter volume) was performed with the 10 ml boluses of 5% hypertonic saline through the distal port of the pulmonary artery catheter during a short period of apnea (Baan et al., 1984). The conductance signals were then converted to calibrated volume signals by considering the inter-electrode spacing, the parallel conductance correction and the CO calibration factor obtained from thermodilution (Baan et al., 1984; Kass et al., 1986). CO calibration and parallel conductance correction were performed before starting the experimental protocol and after the fluid bolus stage.

**FIGURE 1:**
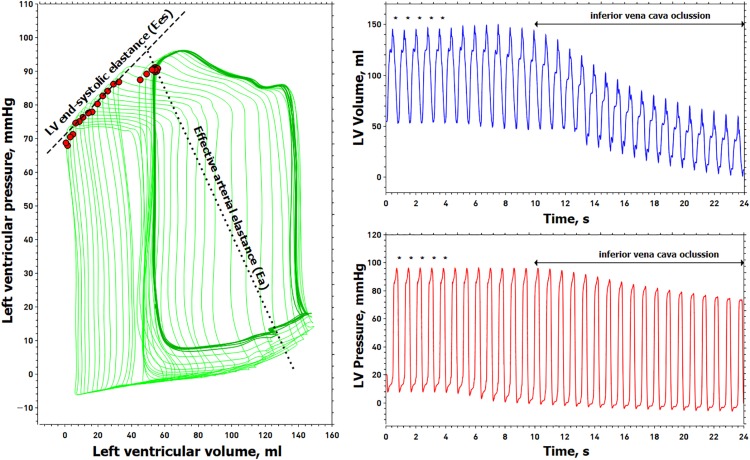
Example of the left ventricular pressure-volume analysis. Left, left ventricular (LV) pressure-volume loops (green line). Right, simultaneous recording of LV volume (blue line) and pressure (red line), from which LV pressure-volume loops were constructed. Red points in the PV loop are the maximal elastance for each cardiac cycle. The slope of these maximum elastance values is obtained during a transient decrease in preload represents the LV end-systolic elastance (Ees). The outlined pressure-volume loops (dark green line) represent five cardiac cycles obtained just before the inferior vena cava (IVC) occlusion (marked with an asterisk above the pressure-volume waveforms) for determining end-systolic pressure, stroke volume, end-diastolic volume, end-systolic volume, and effective arterial elastance (Ea). The dashed line connects the maximal elastance during the IVC maneuver and represents the end-systolic elastance (Ees). The dotted line connects the end-systolic pressure with the end-diastolic volume representing the effective arterial elastance (Ea).

Left ventricle pressure-volume data acquisition and analysis were performed by a dedicated software system (Conduct NT, version 3.18.1, CD Leycom, Zoetermeer, Netherlands). The signals were recorded at 250 Hz and filtered using a 25 Hz low-pass filter. Before and after each experimental stage, three transient occlusions of the inferior vena cava (IVC) were performed using a 25 mm Fogarty balloon (Edwards Transfemoral Balloon Catheter, 9350BC25, Edwards Lifesciences) percutaneously introduced into the right femoral vein. All recordings were done during prolonged apnea in end-expiration. This procedure was repeated if ectopic beats were detected. End-systolic pressure, SV, CO, LV EDV, LV ESV, LV EDP, LVEF, and effective arterial elastance [Ea = Pes/SV, a lumped parameter of LV afterload accounting for pulsatile and resistive load (Sunagawa et al., 1984)] were calculated from 5 beats in steady-state conditions during the respiratory pause just before the IVC occlusion. LV Ees was calculated using the iterative regression method (Kass et al., 1986), as the slope of the ESPVR determined from the linear regression analysis of the maximal E points on each cardiac cycle, defined as E(t) = P(t)/V(t)−V_0,_ where V_0_ is volume-axis intercept or the LV unstressed volume. An example of a PV loop analysis is shown in Figure 1.

A 5-French pigtail catheter was inserted using fluoroscopy through the right carotid or femoral artery into the aortic arch for continuous measurement of central aortic pressure. The radial artery was surgically exposed and cannulated with a 5-French catheter. Arterial pressure waveforms from the ascending aortic and radial arterial artery were continuously recorded with a fluid-filled pressure transducer (FloTracIQ sensor; Edwards Lifesciences) at a sampling rate of 100 Hz, using the EV1000 monitor (Edwards Lifesciences) and then transferred onto a computer. The pressure signals were filtered to remove noise and artifacts using a 10 Hz low pass filter.

### Assessment of Ventriculo-Arterial Coupling and Left Ventricular Efficiency

Ventriculo-arterial coupling was defined as the ratio of Ea to Ees (Sunagawa et al., 1983). LV PV area (PVA) represents the total mechanical energy and was determined as the sum of SW and PE, where SW is the area within the PV loop and PE = 0.5 × Pes × (ESV−V_0_). The ratio of SW/PVA represents the LV_eff_ of converting the total mechanical energy available into SW (Burkhoff and Sagawa, 1986; Asanoi et al., 1989).

### Assessment of Dynamic Arterial Elastance and Arterial Load

Ea_dyn_ was calculated as the ratio of PPV calculated from the aortic pressure waveform and the SVV obtained from the conductance catheter. PPV and SVV were calculated as [(PPmax−PPmin)/(PPmax + PPmin)/2] × 100, and SVV = [(SVmax−SVmin)/(SVmax + SVmin)/2] × 100. Where PPmax and SVmax, and PPmin and SVmin represent the maximal and minimal arterial pulse pressure and stroke volume determined during the same respiratory cycle, respectively. PPV and SVV values were obtained simultaneously and averaged during 1-minute just before the IVC occlusion. A peripheral Ea_dyn_ was also calculated as the ratio between PPV obtained from the radial arterial line and conductance derived SVV. Although arterial load was globally assessed by Ea, we evaluated the contribution of its main components with a two-element Windkessel model of the arterial system, consisting in the net arterial compliance (C_art_ = aortic pulse pressure/stroke volume) and total peripheral resistance [TPR = mean aortic pressure (MAP)/CO] as a measure of total LV pressure load (Segers et al., 2002).

### Experimental Protocol

Animals were initially resuscitated with colloids (Voluven^®^, 130/0.4, Fresenius Kabi Deutschland GmbH, Bad Homburg, Germany) until no further increase in CO. Then they were allowed to stabilize for at least 10 min. Stability was defined as heart rate and MAP variation <5% (Monge Garcia et al., 2018). The study protocol consisted of three consecutive stages with two opposite interventions: changes in afterload (phenylephrine and nitroprusside), preload (bleeding and fluid bolus), and contractility (esmolol and dobutamine). The experiment started with the afterload interventions: animals were treated with a phenylephrine infusion (30–120 mg.kg^–1^.min^–1^) to increase MAP by 40% mmHg from baseline and were allowed to recover at least for 10 min. Then they were treated with sodium nitroprusside (100–200 mg.kg^–1^.min^–1^) to decrease MAP by 40% from baseline but not below 50 mmHg for allowing an adequate hemodynamic tolerance during the IVC occlusions, followed by recovery to baseline status at least for 10 min. Subsequently, for preload interventions, the animals were submitted to stepwise bleeding of 12 ml.kg^–1^ (50 ml.min^–1^) and the blood stored into a heparinized sterile bag. Then the blood was reinfused at the same rate and a fluid bolus of 10 ml.kg^–1^ of a colloid was infused in 5 min. After the fluid administration, the contractility interventions followed: an esmolol infusion was started at 50 μg.kg^–1^.min^–1^ and increased until reducing LV dP/dt_max_ by 50% from its previous value, with a limit dose of 200 μg.kg^–1^.min^–1^. Then the esmolol infusion was stopped and, after a minimum period of 10 min for recovery, the animals were treated with a dobutamine infusion (5 μg.kg^–1^.min^–1^) to increase LV dP/dt_max_ by 50%. LV PV loops and arterial pressure waveforms were obtained at each baseline and after each intervention. At the end of the experiment the animals were euthanized with an intravenous dose (1 ml/4.5 kg) of a euthanasia solution (Euthasol vet, 400 mg/ml: 390 mg pentobarbital sodium and 50 mg phenytoin sodium, Virbac, Reading, Carros, France).

### Statistical Analysis

Data are expressed as mean ± SD. The normality of data was checked by the Shapiro-Wilk test. The intra-observer variability for thermodilution-derived CO measurements and Ees calculation was analyzed with the intraclass correlation coefficient (ICC) with its 95% confidence interval (95% CI), where a ICC > 0.9 represents an excellent agreement between repeated measurements (Koo and Li, 2016). Since we were interested in the effects of individual interventions, differences during each intervention were assessed by a paired *t*-test or a Wilcoxon test. Linear mixed-effects model analysis was used for assessing the relationship between continuous variables, using individual animals as a subject for random factors and sequential experimental stages as repeated measurements. This allowed us to consider the correlation between subjects and non-constant variability over time, which is not considered by the standard linear regression analysis. A heterogeneous first-order autoregressive covariance structure was selected based on the lowest corrected Akaike’s Information Criteria value (Fitzmaurice et al., 2011). Model parameters were estimated via the restricted maximum likelihood method and the estimated fixed effect of each parameter was quantified by using its estimated value (95% CI). Least-squares regression analysis and scatter plots were used for describing the relationship between continuous variables. *P* value < 0.05 was considered statistically significant. Statistical analyses were performed using MedCalc Statistical Software version 19.0.0 (MedCalc Software bvba, Ostend, Belgium; 2019)^1^ and SPPS (SPSS 21, SPPS Inc., Chicago, IL, United States).

## Results

The ICC for thermodilution CO measurements was 0.979 (95%CI: 0.948 to 0.994) at the first calibration and 0.987 (95%CI: 0.965 to 0.996) after fluid administration, respectively. The mean ICC for Ees calculation during all experimental stages was 0.903 (95% CI: 0.822 to 0.985). The mean peak inspiratory was pressure 25 ± 3 cm H_2_O. Blood gas analysis at the beginning of the experiment was: pH 7.53, pCO_2_ 42 mmHg, pO_2_ 149 mmHg, and HCO_3_^–^ 32.2 mmol/L.

### Global Hemodynamic Changes

The evolution of the hemodynamic variables throughout different experimental conditions is detailed in Table 1 (for afterload interventions), Table 2 (for preload interventions), and Table 3 (for contractility interventions). During the afterload interventions, phenylephrine increased MAP by 39 ± 7%, while sodium nitroprusside decreased it by 33 ± 6%. During the preload interventions, bleeding reduced EDV by 10 ± 8%, while reinfusion and fluid administration increased it by 11 ± 21%. In both cases, neither CO nor SV were significantly modified. During contractility interventions, esmolol infusion decreased LV dPdt_max_ by 49 ± 16%, while dobutamine increased it by 89 ± 23%.

**TABLE 1:** Hemodynamic variables during afterload changes.

	**Phenylephrine**			**Sodium Nitroprusside**		
	**Before**	**After**	*p* value*	**Before**	**After**	*p* value*
**Global hemodynamics**						
CO, l⋅min^–1^	7.81 (1.31)	7.03 (0.97)	0.001	8.00 (1.53)	8.85 (1.46)	0.014
SV, ml	106 (10)	96 (11)	<0.001	101 (11)	119 (14)	<0.001
HR, beats⋅min^–1^	74 (10)	73 (8)	0.725	79 (11)	75 (12)	0.054
MAP, mmHg	79 (11)	109 (14)	<0.001	81 (8)	55 (4)	<0.001
PPV,%	12 (2)	7 (1)	<0.001	12 (2)	20 (7)	0.002
SVV,%	9 (3)	9 (4)	0.972	10 (4)	12 (5)	0.072
Ea_dyn_	1.51 (0.44)	0.98 (0.38)	<0.001	1.35 (0.38)	1.79 (0.57)	<0.001
**LVhemodynamics**						
EDV, ml	209 (48)	207 (45)	0.461	202 (44)	204 (47)	0.533
ESV, ml	104 (42)	111 (40)	0.152	101 (40)	85 (43)	<0.001
Ped, mmHg	11 (5)	17 (5)	<0.001	11 (4)	6 (4)	<0.001
Pes, mmHg	82 (12)	119 (15)	<0.001	89 (11)	59 (7)	<0.001
Ejection fraction,%	52 (10)	48 (9)	0.011	52 (10)	61 (12)	<0.001
dP/dt_max_, mmHg⋅s^–1^	1004 (183)	1231 (172)	<0.001	1096 (164)	974 (193)	0.001
**Arterial vascular mechanics**						
TPR, dyn⋅ < *cps*:*it* > *cm* < /*cps*:*it* > ⋅s^–5^	827 (182)	1257 (199)	<0.001	834 (154)	504 (92)	<0.001
C_art_, ml⋅mmHg^–1^	3.93 (0.90)	2.81 (0.56)	<0.001	3.22 (0.69)	4.53 (0.83)	<0.001
**Ventriculo-arterial coupling and LV mechanical efficiency**						
Ees, mmHg⋅ml^–1^	0.38 (0.14)	0.46 (0.11)	0.035	0.42 (0.13)	0.42 (0.20)	0.863
Ea, mmHg⋅ml^–1^	0.79 (0.16)	1.26 (0.24)	<0.001	0.89 (0.16)	0.50 (0.09)	<0.001
VAC (Ea/Ees)	2.25 (0.71)	2.83 (0.60)	0.004	2.25 (0.55)	1.45 (0.57)	<0.001
SW, mmHg⋅ml	6994 (1220)	8186 (1274)	<0.001	7176 (1438)	6713 (1394)	0.014
PE, mmHg⋅ml	4213 (1634)	6588 (2421)	<0.001	4382 (1501)	2407 (1020)	<0.001
LV_eff_,%	63.4 (9.9)	56.6 (9.9)	<0.001	62.5 (10.3)	73.7 (11.1)	<0.001

Values are presented as means (SD). * Paired *t*-test for before and after each experimental intervention. LV, left ventricle; CO, cardiac output; SV, stroke volume; HR, heart rate; MAP, mean arterial pressure; EDV, left ventricular end-diastolic volume; ESV, left ventricular end-systolic volume; Ped, left ventricular end-diastolic pressure; Pes, left ventricular end-systolic pressure; PPV, pulse pressure variation; SVV, stroke volume variation; Ea_*dyn*_, dynamic arterial elastance; dP/dt_*max*_, peak rate of left ventricular pressure; TPR, total peripheral resistance; C_*art*_, arterial compliance; Ees, end-systolic elastance; Ea, effective arterial elastance; VAC, ventriculo-arterial coupling ratio; SW, left ventricular stroke work; PE, left ventricular potential energy; LV_*eff*_, left ventricular mechanical efficiency.

**TABLE 2:** Hemodynamic variables during preload changes.

	**Bleeding**			**Fluid loading**		
	**Before**	**After**	*p* value*	**Before**	**After**	*p* value*
**Global hemodynamics**						
CO, l⋅min^–1^	8.06 (1.65)	7.92 (1.57)	0.664	8.06 (1.84)	8.99 (3.01)	0.142
SV, ml	109 (17)	111 (18)	0.592	110 (18)	116 (31)	0.243
HR, beats⋅min^–1^	74 (10)	72 (13)	0.347	73 (11)	77 (10)	0.029
MAP, mmHg	79 (12)	56 (7)	<0.001	64 (8)	79 (12)	0.007
PPV,%	14 (3)	24 (6)	<0.001	21 (6)	10 (4)	0.001
SVV,%	10 (5)	17 (7)	0.001	15 (7)	8 (4)	0.006
Ea_dyn_	1.58 (0.56)	1.53 (0.48)	0.681	1.50 (0.39)	1.42 (0.64)	0.551
**LV hemodynamics**						
EDV, ml	225 (52)	203 (56)	0.001	207 (52)	247 (54)	<0.001
ESV, ml	115 (48)	92 (49)	<0.001	97 (47)	131 (46)	<0.001
Ped, mmHg	11 (3)	4 (4)	<0.001	7 (4)	15 (4)	<0.001
Pes, mmHg	82 (13)	61 (8)	<0.001	71 (8)	81 (11)	0.015
Ejection fraction,%	50 (11)	57 (12)	<0.001	55 (12)	48 (11)	0.008
dP/dt_max_, mmHg⋅s^–1^	1008 (192)	862 (228)	<0.001	947 (199)	1020 (157)	0.117
**Arterial vascular mechanics**						
TPR, dyn⋅ < *cps*:*it* > *cm* < /*cps*:*it* > ⋅s^–5^	805 (194)	580 (119)	<0.001	662 (178)	768 (280)	0.187
C_art_, ml⋅mmHg^–1^	4.37 (0.93)	5.00 (1.19)	0.001	4.61 (0.92)	4.72 (1.01)	0.384
**Ventriculo-arterial coupling and LV mechanical efficiency**						
Ees, mmHg⋅ml^–1^	0.40 (0.14)	0.41 (0.16)	0.663	0.42 (0.16)	0.32 (0.12)	0.003
Ea, mmHg⋅ml^–1^	0.77 (0.20)	0.57 (0.12)	<0.001	0.66 (0.14)	0.78 (0.36)	0.182
VAC (Ea/Ees)	2.18 (0.84)	1.58 (0.59)	<0.001	1.79 (0.77)	2.58 (0.96)	0.002
SW, mmHg⋅ml	6891 (1435)	6047 (1035)	0.028	6609 (1244)	7522 (1833)	0.018
PE, mmHg⋅ml	4599 (1703)	2715 (1260)	<0.001	3364 (1524)	5349 (1917)	0.001
LV_eff_,%	60.6 (11.7)	69.7 (11.4)	<0.001	67 (12.1)	58.9 (12)	0.002

Values are presented as means (SD). * Paired *t*-test for before and after each experimental intervention. LV, left ventricle; CO, cardiac output; SV, stroke volume; HR, heart rate; MAP, mean arterial pressure; EDV, left ventricular end-diastolic volume; ESV, left ventricular end-systolic volume; Ped, left ventricular end-diastolic pressure; Pes, left ventricular end-systolic pressure; PPV, pulse pressure variation; SVV, stroke volume variation; Ea_dyn_, dynamic arterial elastance; dP/dt_max_, peak rate of left ventricular pressure; TPR, total peripheral resistance; C_art_, arterial compliance; Ees, end-systolic elastance; Ea, effective arterial elastance; VAC, ventriculo-arterial coupling ratio; SW, left ventricular stroke work; PE, left ventricular potential energy; LV_eff_, left ventricular mechanical efficiency.

**TABLE 3:** Hemodynamic variables during contractility changes.

	**Esmolol**			**Dobutamine**		
	**Before**	**After**	*p* value*	**Before**	**After**	*p* value*
**Global hemodynamics**						
CO, l⋅min^–1^	9.67 (2.31)	6.02 (1.88)	<0.001	8.37 (2.06)	11.74 (3.41)	<0.001
SV, ml	127 (25)	88 (25)	<0.001	114 (19)	136 (27)	<0.001
HR, beats⋅min^–1^	78 (11)	70 (9)	<0.001	73 (10)	85 (14)	<0.001
MAP, mmHg	72 (13)	51 (4)	<0.001	71 (9)	85 (11)	<0.001
PPV,%	11 (4)	14 (3)	<0.001	11 (4)	11 (4)	0.563
SVV,%	7 (3)	15 (6)	<0.001	8 (2)	6 (2)	0.006
Ea_dyn_	1.66 (0.50)	1.06 (0.41)	<0.001	1.39 (0.48)	1.77 (0.58)	0.009
**LV hemodynamics**						
EDV, ml	223 (40)	221 (45)	0.530	225 (33)	222 (32)	0.102
ESV, ml	96 (34)	134 (36)	<0.001	109 (26)	83 (30)	<0.001
Ped, mmHg	14 (4)	12 (3)	0.028	14 (4)	15 (4)	0.035
Pes, mmHg	74 (15)	54 (8)	<0.001	74 (12)	87 (14)	<0.001
Ejection fraction,%	57 (9)	40 (9)	<0.001	51 (8)	63 (11)	<0.001
dP/dt_max_, mmHg⋅s^–1^	1067 (209)	528 (156)	<0.001	929 (159)	1756 (377)	<0.001
**Arterial vascular mechanics**						
TPR, dyn⋅ < *cps*:*it* > *cm* < /*cps*:*it* > ⋅s^–5^	635 (216)	737 (240)	0.045	721 (215)	628 (224)	<0.001
C_art_, ml⋅mmHg^–1^	4.69 (1.05)	4.02 (1.05)	<0.001	4.46 (0.93)	4.63 (0.99)	0.005
**Ventriculo-arterial coupling and LV mechanical efficiency**						
Ees, mmHg⋅ml^–1^	0.36 (0.11)	0.24 (0.07)	<0.001	0.30 (0.07)	0.48 (0.14)	<0.001
Ea, mmHg⋅ml^–1^	0.63 (0.19)	0.67 (0.18)	0.248	0.67 (0.18)	0.67 (0.19)	0.764
VAC (Ea/Ees)	1.73 (0.44)	2.78 (0.55)	<0.001	2.28 (0.69)	1.50 (0.59)	<0.001
SW, mmHg⋅ml	7421 (1217)	3443 (1623)	<0.001	6640 (1131)	10199 (2120)	<0.001
PE, mmHg⋅ml	3514 (1201)	3505 (793)	0.975	3999 (1103)	3609 (1348)	0.039
LV_eff_,%	68.1 (9.1)	48.2 (10.2)	<0.001	62.6 (7.6)	73.6 (9.8)	<0.001

Values are presented as means (SD). * Paired *t*-test for before and after each experimental intervention. LV, left ventricle; CO, cardiac output; SV, stroke volume; HR, heart rate; MAP, mean arterial pressure; EDV, left ventricular end-diastolic volume; ESV, left ventricular end-systolic volume; Ped, left ventricular end-diastolic pressure; Pes, left ventricular end-systolic pressure; PPV, pulse pressure variation; SVV, stroke volume variation; Ea_dyn_, dynamic arterial elastance; dP/dt_max_, peak rate of left ventricular pressure; TPR, total peripheral resistance; C_art_, arterial compliance; Ees, end-systolic elastance; Ea, effective arterial elastance; VAC, ventriculo-arterial coupling ratio; SW, left ventricular stroke work; PE, left ventricular potential energy; LV_eff_, left ventricular mechanical efficiency.

### Ventriculo-Arterial Coupling and Left Ventricular Efficiency

**FIGURE 2:**
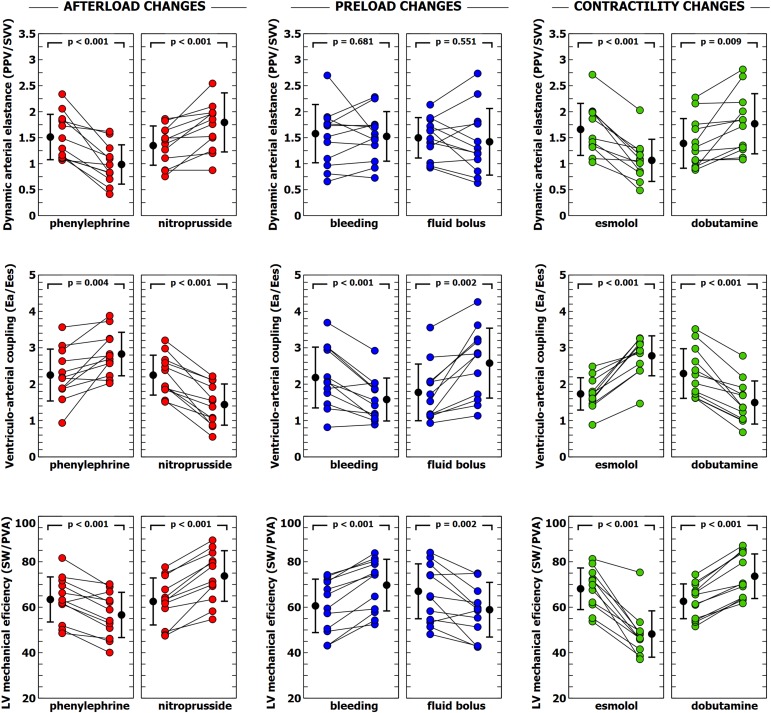
Evolution of dynamic arterial elastance, ventricular-arterial coupling and left ventricular efficiency during different experimental interventions. Individual values of dynamic arterial elastance (Ea_dyn_), ventricular-arterial coupling (Ea/Ees) and left ventricular mechanical efficiency during each experimental stage. Black points with bars represent the mean value and the standard deviation. Colored points represent individual changes for each animal.

**FIGURE 3:**
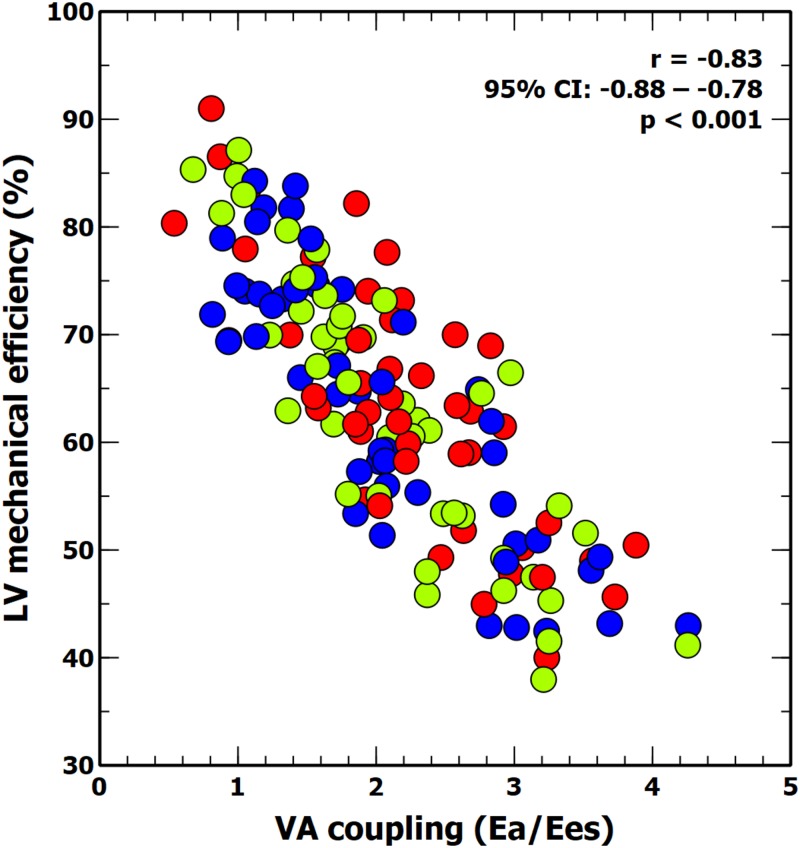
Scatter plot: linear correlation between ventricular-arterial (VA) coupling and LV mechanical efficiency. Colors inside circles represent different experimental interventions: red, afterload; green: preload; blue: contractility.

The individual changes in VA coupling and LV_eff_ during each experimental stage are shown in Figure 2. All experimental interventions affected to a different extent both VA coupling and LV_eff_. These changes were related to the relative variations on Ea and Ees. Afterload interventions mainly modified VA coupling by altering Ea (Table 1), contractility interventions changed VA coupling by isolated variations in Ees (Table 3). Preload modifications, however altered VA coupling in different ways: bleeding decreased VA coupling by reducing Ea, while fluid administration increased VA coupling by decreasing Ees without changing Ea (Table 2). As expected, there was an inverse relationship between VA coupling and LV_eff_ (Figure 3). The maximal LV_eff_ was associated with lower VA coupling values.

### Evolution of Ea_dyn_, PVV, and SVV

**FIGURE 4:**
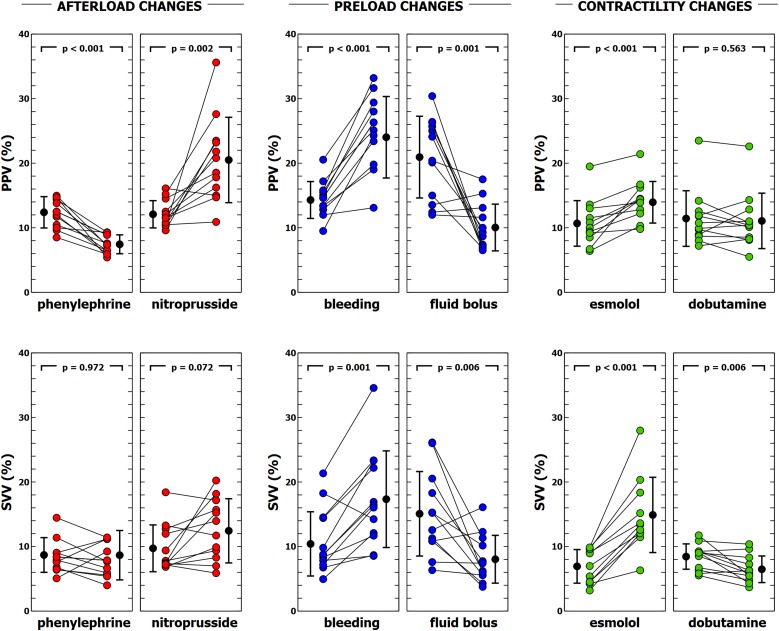
Evolution of pulse pressure variation (PPV) and stroke volume variation (SVV) during different experimental interventions. Individual values of PPV and SVV during each experimental condition. Black points with bars represent the mean value and the standard deviation. Colored points represent individual changes for each animal.

Individual changes in Ea_dyn_, PPV, and SVV are shown in Figures 2, 4. Ea_dyn_ was significantly modified during afterload and contractility changes, whereas it remained unchanged after bleeding and fluid administration. Sodium nitroprusside increased Ea_dyn_, while phenylephrine decreased it. On the other hand, an improvement in contractility with dobutamine increased Ea_dyn_, while reducing contractility with esmolol decreased Ea_dyn_. The overall correlation between SVV and PPV during the whole study was *r* = 0.64 (95% CI: 0.533 to 0.729; *p* < 0.001). Radial and aortic PPV were highly correlated (*r* = 0.92; 95% CI: 0.890 to 0.941; *p* < 0.001). There was a significant relationship between PPV (but not SVV) and arterial system properties: a low TPR value and high compliance were associated with a higher PPV (Table 4).

**TABLE 4:** Relationship between arterial vascular mechanics on pulse pressure variation (PPV) and stroke volume variation (SVV) according to a mixed-effect regression model.

	**PPV**		**SVV**	
**Fixed effect**	**Estimate (95% CI)**	***p* value**	**Estimate (95% CI)**	***p* value**
TPR, dyn⋅ < *cps*:*it* > *cm* < /*cps*:*it* > ⋅s^–5^	−0.007 (−0.010 to −0.005)	<0.001	0.001 (−0.002 to 0.004)	0.562
C_art_, ml⋅mmHg^–1^	0.961 (0.125 to 1.798)	0.025	−0.342 (−1.301 to 0.618)	0.480

*Estimates reflect the average change in PPV or SVV per unit increase of the fixed effect. PPV, pulse pressure variation; SVV, stroke volume variation; CI, confidence interval; TPR, total peripheral resistance; C_art,_ arterial compliance.*

### Effects of Ventriculo-Arterial Coupling and Left Ventricular Efficiency on PPV, SVV, and Ea_dyn_

**FIGURE 5:**
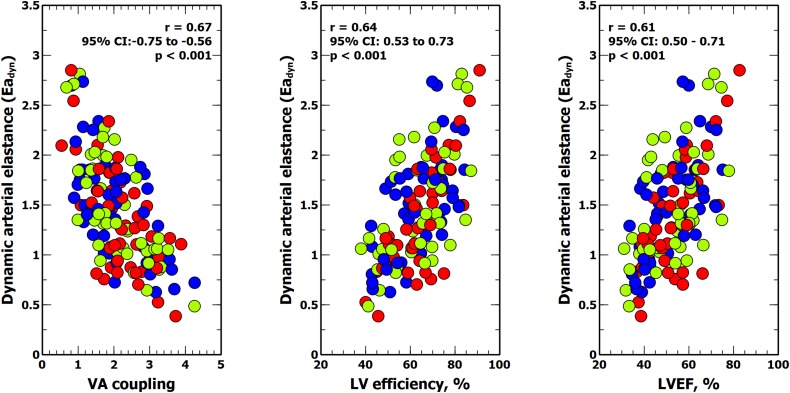
Relationship between dynamic arterial elastance, ventricular-arterial (VA) coupling, left ventricular (LV) mechanical efficiency and left ventricular ejection fraction (LVEF). Colors inside circles represent different experimental interventions: red, afterload; green: preload; blue: contractility.

Table 5 shows the impact of VA coupling and LV_eff_ on PVV, and SVV. While PPV was only inversely related to Ea, SVV was directly associated with Ees. Ea_dyn_ was significantly related to VA coupling, LV_eff_ and its determinants (Table 6 and Figure 5). Changes in both Ea and Ees and were associated with changes in Ea_dyn_. A 10% decrease in Ea increased Ea_dyn_ by 0.09, while a similar change in Ees was associated with an Ea_dyn_ reduction of 0.22. Accordingly, the lower the VA coupling and the better the LV_eff_, the higher the Ea_dyn_. This relationship remained when using radial PPV for calculating Ea_dyn_ (Table 7), including a significant relationship between Ea_dyn_ and LVEF (estimate: 0.026, 95% CI: 0.021 to 0.032, *p* < 0.001).

**TABLE 5:** Estimated values of fixed effects on aortic pulse pressure variation (PPV) and stroke volume variation (SVV) according to a mixed-effect regression model.

	**PPV**		**SVV**	
**Fixed effect**	**Estimate (95% CI)**	***p* value**	**Estimate (95% CI)**	***p* value**
**Ventriculo-arterial coupling indexes**				
VAC (Ea/Ees)	−0.041 (−0.877 to 0.795)	0.923	2.321 (1.801 – 2.841)	<0.001
Ees, mmHg⋅ml^–1^	−2.653 (−7.589 to 2.284)	0.288	−6.922 (−10.567 to −3.278)	<0.001
Ea, mmHg⋅ml^–1^	−7.729 (−10.130 to −5.327)	<0.001	−0.060 (−1.972 to 2.093)	0.953
**Left ventricular energetics**				
LV_eff_,%	−0.044 (−0.092 to 0.004)	0.069	−0.143 (−0.187 to −0.100)	<0.001
Stroke work, mmHg⋅l	−0.617 (−0.846 to −0.388)	<0.001	−0.560 (−0.768 to −0.362)	<0.001
Potential energy mmHg⋅l	−0.870 (−1.210 to −0.530)	<0.001	0.067 (−0.227 to 0.362)	0.650

*Estimates reflect the average change in PPV or SVV per unit increase of the fixed effect. PPV, pulse pressure variation; SVV, stroke volume variation; CI, confidence interval; Ees, end-systolic elastance; Ea, effective arterial elastance; VAC, ventriculo-arterial coupling; LV_eff_, left ventricular mechanical efficiency.*

**TABLE 6:** Relationship between ventriculo-arterial coupling and left ventricular efficiency and Ea_dyn_ according to a mixed-effect regression model.

		**95% confidence**	
**Fixed effects**	**Estimate**	**interval**	***p* value**
**Ventriculo-arterial coupling indexes**			
VAC (Ea/Ees)	–0.414	−0.486 to −0.343	<0.001
Ees, mmHg⋅ml^–1^	2.271	1.766 to 2.776	<0.001
Ea, mmHg⋅ml^–1^	–0.933	−1.196 to −0.671	<0.001
**Left ventricular energetics**			
LV_eff_,%	0.026	0.021 to 0.031	<0.001
Stroke work, mmHg⋅l	0.107	0.079 to 0.134	<0.001
Potential energy mmHg⋅l	–0.121	−0.160 to −0.082	<0.001

*Estimates reflect the average change in Ea_dyn_ per unit increase of the fixed effect. Ea_dyn_, dynamic arterial elastance; Ees, left ventricular end-systolic elastance; Ea, effective arterial elastance; VAC, ventriculo-arterial coupling; LV_eff_, left ventricular mechanical efficiency.*

**TABLE 7:** Relationship between ventriculo-arterial coupling and left ventricular efficiency and peripheral Ea_dyn_ (radial PPV/SVV) according to a mixed-effect regression model.

		**95% confidence**	
**Fixed effects**	**Estimate**	**interval**	***p* value**
**Ventriculo-arterial coupling indexes**			
VAC (Ea/Ees)	–0.380	−0.460 to −0.300	<0.001
Ees, mmHg⋅ml^–1^	2.259	1.742 to 2.776	<0.001
Ea, mmHg⋅ml^–1^	–0.888	−1.166 to −0.609	<0.001
**Left ventricular energetics**			
LV_eff_,%	0.022	0.016 to 0.027	<0.001
Stroke work, mmHg⋅l	0.083	0.051 to 0.116	<0.001
Potential energy mmHg⋅l	–0.112	−0.152 to −0.072	<0.001

*Estimates reflect the average change in peripheral Ea_dyn_ per unit increase of the fixed effect. Ea_dyn_, dynamic arterial elastance; PPV, pulse pressure variation; SVV, stroke volume variation; Ees, left ventricular end-systolic elastance; Ea, effective arterial elastance; VAC, ventriculo-arterial coupling; LV_eff_, left ventricular mechanical efficiency.*

## Discussion

In this study, we aimed to determine the relationship between Ea_dyn_, VA coupling and LV mechanical efficiency while altering loading and contractile conditions in anesthetized healthy pigs. Our conclusions are twofold. First, Ea_dyn_ changes were related to both afterload and LV contractility. The relative contributions of these two factors to both PPV and SVV eventually lead to the observed Ea_dyn_ value. These findings agree with previous reports and confirm the compound nature of Ea_dyn_ (Monge Garcia et al., 2017a, b). Consequently, Ea_dyn_ does not behave as a true measure of LV afterload nor an index of vasomotor tone, but rather a variable resulting from the interaction between LV contractility and the arterial system. Second, it follows therefore that because of the impact of both LV systolic function (Ees) and arterial system properties (Ea), a significant relationship between Ea_dyn_ and VA coupling exists with higher Ea_dyn_ associated with better VA coupling. Moreover, since the LV oxygen consumption required for a given SW depends on both loading conditions and myocardial contractile state (Burkhoff and Sagawa, 1986), Ea_dyn_ was also associated with LV mechanical efficiency. Accordingly, Ea_dyn_ increases with improvements in VA coupling and LV_eff_ and decreases when cardiovascular performance and LV mechanical efficiency worsen.

### The Nature of Ea_dyn_

By definition, the nature of Ea_dyn_ is determined by how respiration altered both LV SV and arterial pulse pressure. Briefly, respiratory variations in LV SV are the consequence of the cyclic changes in intrathoracic pressure and lung volumes on both right and left ventricular preload and afterload. The magnitude of these variations defines the preload-dependency and depicts the slope of the cardiac function curve (Pinsky, 2012). Considering that arterial pressure is a function of LV outflow and arterial load (Sunagawa et al., 1983), variations in arterial pulse pressure should mainly reflect LV SV changes as long as both arterial system properties and LV ejection pattern remain unchanged (Pinsky, 2005). Therefore, PPV is mostly determined by SVV at a given time, making PPV a valid surrogate for assessing preload-dependency (Marquez et al., 2008; Pinsky, 2012) because variations in the arterial system and LV function during a respiratory cycle are usually negligible (Pinsky, 1997). However, significant changes in contractility and arterial load may occur over longer periods of time. Moreover, clinical conditions such as the use of vasoactive therapy (Kubitz et al., 2008; Renner et al., 2009; Hadian et al., 2011; Monge Garcia et al., 2017a; de Courson et al., 2019; Wodack et al., 2019), acute hemorrhage (Berkenstadt et al., 2005; Renner et al., 2009), fluid administration (Kim and Pinsky, 2008; Monge Garcia et al., 2011; Cecconi et al., 2014; Monge Garcia et al., 2014; Seo et al., 2015), sepsis (Cherpanath et al., 2014) or pharmacological changes in contractility (Mesquida et al., 2011), may alter the interaction between PPV and SVV and therefore modify the eventual value of Ea_dyn_.

**FIGURE 6:**
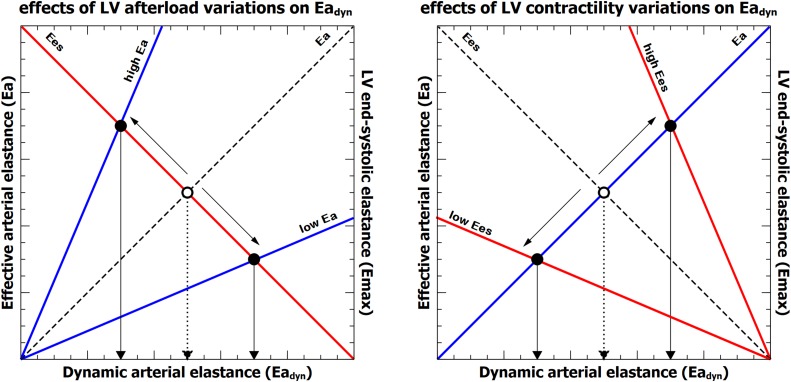
Representation of the relationship between effective arterial elastance (Ea, blue line), left ventricular end-systolic elastance (Ees, red line) and dynamic arterial elastance (Ea_dyn_). Left: effects of isolated variations on left ventricular afterload (Ea) on Ea_dyn_. Right, effects of isolated changes of left ventricular contractility (Ees). The dashed line and open circle represent the baseline condition. The intersection between Ea and Ees defines the value of Ea_dyn_ on the x-axis for each ventriculo-arterial coupling (Ea/Ees) condition.

In our study, PPV was significantly associated with changes in Ea and related to both TPR and C_art_, acting mostly as a peripheral factor, while SVV was associated with Ees, representing more as the central component of Ea_dyn_. Thus, the nature of Ea_dyn_ was defined by the interaction between the arterial system and the LV systolic function: while increases in afterload decreases Ea_dyn_, an increase in LV systolic function also increases Ea_dyn_ (Figure 6). Consequently, a lower Ea/Ees ratio, i.e., a better VA coupling, would therefore be associated to a higher Ea_dyn_ value, while a worse cardiovascular performance (higher VA coupling value) would be related to a lower Ea_dyn_ value. Moreover, as LV mechanical efficiency depends on the interaction of the arterial load and LV function (Sunagawa et al., 1985; Burkhoff and Sagawa, 1986), we also found a positive relationship between Ea_dyn_ and LV_eff_. The higher the Ea_dyn_, the higher LV_eff_. So, at higher Ea_dyn_ values the interaction between the LV and the arterial system is associated to a better LV mechanical efficiency, while a decrease in Ea_dyn_ would represent a higher energy consumed by the heart to achieve the required SW (lower LV_eff_).

### Ea_dyn_ for Guiding Fluid Therapy in Hypotensive and Preload-Dependent Patients

The first application of the physiological concept of Ea_dyn_ on the clinical setting was as an index for predicting the arterial pressure response to fluid administration (Pinsky, 2002; Monge Garcia et al., 2014). The reason behind this idea is that, if variations in SV during mechanical ventilation are followed by proportional or greater changes in arterial pulse pressure, then the likelihood of increasing blood pressure when CO is increased with fluids will be high. On the contrary, if these SV changes are followed by a smaller arterial pulse pressure variations, then the fluid-induced increase in CO will be not followed by an increase in arterial pressure (Pinsky, 2002). In 25 hypotensive and preload-dependent patients receiving a fluid bolus because of acute circulatory failure (Monge Garcia et al., 2011) demonstrated that Ea_dyn_ value obtained from arterial waveform analysis was higher in patients that responded increasing both CO and MAP. This same group, using two independent techniques for estimating Ea_dyn_ (esophageal Doppler for measuring SVV and radial arterial pressure for PPV), confirmed these findings in 53 mechanically ventilated, preload-dependent patients with acute circulatory failure (Monge Garcia et al., 2014): an Ea_dyn_ ≥ 0.73 predicted the subsequent arterial pressure response to fluid administration with a sensitivity of 91% and a specificity of 92%. Seo et al. (2015) reported similar results in 39 patients during robot-assisted laparoscopic prostatectomy: an Ea_dyn_ value of 0.74 predicted a MAP increase ≥ 15% after fluid challenge with a sensitivity of 70.6% and a specificity of 86.4%. Vos et al. (2013) in 30 patients undergoing major hepatic resection, demonstrated that Ea_dyn_ allowed to predict the reduction in norepinephrine requirement at the end of fluid administration due to a significant increase in MAP. More recently, Guarracino et al. (2019) confirmed the usefulness of Ea_dyn_ for predicting the associate change in MAP in response to a CO increase after intravenous fluid infusion in septic patients, suggesting that resuscitation guided by Ea_dyn_ may result in a more rational and efficient fluid resuscitation. Under this premise, the Ea_dyn_ has been also recently included as part of hemodynamic optimization protocol for reducing intraoperative hypotension (Davies et al., 2019; Maheshwari et al., 2019).

In all previous studies, an Ea_dyn_ value > 1 usually represents the ability of increasing both arterial and CO with fluid administration in patients with acute circulatory shock. According to the current results, a high Ea_dyn_ value should be associated with a better VA coupling and LV_eff_. Therefore, in a hypotensive and preload-dependent patient, an Ea_dyn_ value > 1 would reflect a cardiovascular system able to increase pressure with flow changes while moving from a better LV_eff_ (lower energy consumption) to a higher SW status (better mechanical efficacy) (Burkhoff and Sagawa, 1986; Asanoi et al., 1989). Thus, a better VA coupling and LV_eff_ conditions would provide favorable conditions for increasing both CO and arterial pressure with fluids. That reasoning would also explain the reduction in Ea_dyn_ observed after fluid administration (Kim and Pinsky, 2008; Monge Garcia et al., 2011, 2014; Cecconi et al., 2014; Seo et al., 2015). Kim and Pinsky (2008) reported a decrease in PPV/SVV ratio from 1.47 (PPV 18.2%; SVV 12.4%) to 1.03 (PPV 7.7%; SVV 7.5%) in seven pentobarbital-anesthetized intact dogs submitted to volume loading. These results agreed with those reported in the clinical studies testing the usefulness of Ea_dyn_ in hypotensive patients. In the first study of Monge Garcia et al. (2011) Ea_dyn_ decreased from 1.34 ± 0.45 to 0.85 ± 0.21 in the group of patients with a significant increase in pressure, whereas it remained unchanged in patients with no change in arterial pressure. A similar observation was made in later studies (Monge Garcia et al., 2014; de Courson et al., 2019). As Ea_dyn_ parallels LV_eff_, a reduction in Ea_dyn_ should therefore reflect the transition from a higher LV_eff_ to a better LV mechanical efficacy (Asanoi et al., 1989).

Importantly, although Ea_dyn_ appears to be related to the mechanical performance of the coupling between the left ventricle and the arterial circulation, the ability to increase pressure with fluids will depend eventually on the biventricular preload-dependency. If changes in preload are not followed by an increase in CO, then an increase in arterial pressure should not be expected (Monge Garcia et al., 2014). Moreover, as the arterial pressure is tightly regulated, if the reflex pressure control mechanisms are intact, changes in CO should not be followed by significant variations in arterial pressure in normotensive patients (Kubota et al., 1992). However, in those situations in which arterial pressure regulation has been lost, as during hypotension in circulatory shock (Kato and Pinsky, 2015), an increase in CO would result in a rise in arterial pressure depending on the baseline VA coupling. Consequently, the usefulness of Ea_dyn_ for predicting arterial pressure response to fluid administration is conditioned by the presence of biventricular preload-dependency and arterial pressure homeostasis regulatory mechanisms.

There have also been negative studies about the usefulness of Ea_dyn_ for predicting arterial pressure response to fluid administration. These studies mainly failed because Ea_dyn_ was erroneously interpreted as a measure of fluid responsiveness or used in unselected populations. For example, in the study of Stens J et al., Ea_dyn_ was evaluated non-invasively with a finger plethysmography device (Nexfin^®^) for predicting preload-responsiveness after positioning the patient in Trendelenburg position. They found that the degree of fluid responsiveness was not associated with baseline Ea_dyn_ values (Stens et al., 2016). Since Trendelenburg maneuvers will increase arterial pressure independent of flow, such negative results are predictable. In 51 patients scheduled for neurosurgery or elective abdominal surgery, Lanchon et al. (2017) showed that Ea_dyn_ was unable to predict the MAP increase after volume expansion. However, their analysis was performed without prejudging prior preload-responsiveness and baseline MAP was not reported. If their patients were not hypotensive, then one would not expect arterial pressure to increase if CO also increased. Wu et al. (2016) reported low performance of Ea_dyn_ for assessing the arterial pressure response after an intraoperative fluid challenge in 31 liver cirrhosis patients receiving a living donor liver transplant. However, the baseline MAP for preload-dependent patients was 75 (65 to 81 mmHg), which indicates that arterial pressure was already optimized, and the vascular system was likely operating to keep constant pressure, as reflected in the modest MAP increase (from 75 to 77 mmHg) in those 23 patients with a significant increase in SV.

### Ea_dyn_ for Guiding Norepinephrine Eeaning

Guinot et al. (2015, 2017) demonstrated that Ea_dyn_ was able to predict the MAP decrease associated with a reduction in norepinephrine dosage and, therefore, it could be used as an aid for titrating this vasoactive therapy in patients with septic shock or during vasoplegic syndrome following cardiac surgery. Patients with an Ea_dyn_ value > 0.94 were able to sustain MAP despite a decrease in vasopressor support, while patients with a lower Ea_dyn_ significantly reduced MAP after decreasing norepinephrine dose. These authors argued that Ea_dyn_ can be considered as a functional assessment of arterial tone. Considering the current results, a patient with a higher Ea_dyn_ should have better VA coupling and be able to generate an increase in SW that keeps MAP despite the decrease in the external vasopressor support and potentially vasomotor tone. Moreover, these patients showed a decrease in Ea_dyn_ after reducing norepinephrine dose, supporting the hypothesis that the cardiovascular system shifted from a better LV_eff_ and to a higher mechanical efficacy for sustaining MAP. In concordance with this hypothesis (Guarracino et al., 2019) have recently shown that Ea_dyn_ allowed predicting the arterial pressure response to a norepinephrine infusion in patients with septic shock. An Ea_dyn_ value > 0.83, which was associated with a better LV_eff_ and VA coupling conditions estimated by the single beat method and standard echocardiography, allowing them to predict which patients would increase MAP > 15% after the introduction of norepinephrine(Guarracino et al., 2019).

### Ea_dyn_ During Isolated Changes in Arterial Load

Monge Garcia et al. (2017a) reported the changes in Ea_dyn_ during isolated variations in arterial load induced by sodium nitroprusside and phenylephrine in rabbits. In this study, Ea_dyn_ behaved in a similar way to that observed in the current study. Ea_dyn_ increased during sodium nitroprusside-induced vasodilation and decreased during vasoconstriction with the phenylephrine infusion. These findings are also in agreement with the study of Wodack et al. (2019), in which MAP was stepwise increased with phenylephrine and Ea_dyn_ was reduced from 1.06 (0.53) to 0.68 (0.3). Furthermore, in a recent multicenter study (de Courson et al., 2019) have shown that the use of a phenylephrine infusion in 56 surgical patients induced a significant decrease in Ea_dyn_ from 0.67 (0.48–0.80) to 0.54 (0.37–0.68). Therefore, our results underscore the message that Ea_dyn_ does not represent an index of arterial vasomotor tone nor arterial load, although changes in arterial load may affect Ea_dyn_. The unspoken other conclusion of these findings is that phenylephrine is a poor cardiovascular treatment for sustained hypotension because of its detrimental effects on Ea_dyn_ and LV_eff_.

### Ea_dyn_ During Isolated Changes in Contractility

Only a few studies have directly addressed the impact of contractility changes in the PPV and SVV relationship. Mesquida et al. (2011) studied the effects of propranolol-induced acute ventricular failure on the relation between PPV and SVV in thoracotomized mongrel dogs. According to their results, propranolol infusion decreases Ea_dyn_ from 1.16 (PPV 37 ± 24%, SVV 32 ± 16%) to 0.96 (PPV 26 ± 13%, SVV 27 ± 11%). These findings agree with those observed in the sequential changes in PPV and SVV observed by Kim and Pinsky (2008). In their study, the relationship between PPV and SVV was successively altered in anesthetized dogs with esmolol, dobutamine and fluid administration. While fluid administration and esmolol infusion were associated with low PPV/SVV ratios, dobutamine increased Ea_dyn_. Interestingly, the effect of increasing tidal volume from 5 ml/kg to 20 ml/kg also augmented this ratio.

### Ea_dyn_ as a Ventriculo-Arterial Coupling Index

Although the concept of the coupling between the heart and the arterial system has been described for more than 30 years, it has resurged recently in the critical care settings (Guarracino et al., 2013, 2014). This concept offers several potential advantages for the analysis of the cardiovascular system: it defines the behavior of the heart and the arterial system as an interconnected system and not as isolated structures, but it also provides a valid method for evaluating the cardiovascular performance and cardiac energetics relating both cardiac and vascular peripheral functions (Kass and Kelly, 1992).

The cardiovascular system aims to deliver adequate blood flow and pressure to sustain normal physiological functions. Flow is important to deliver oxygen whereas pressure is important to allow for autoregulation of blood flow distribution. Because the interaction of arterial system with the heart, a given arterial pressure and CO that maintains necessary organ perfusion can be achieved by different cardiovascular configurations (Monge Garcia et al., 2016). However, an optimal combination of cardiac function and arterial system state has been described that ensures the maximal mechanical energy transfer from the ventricle to the arterial tree (Guarracino et al., 2013). Previous studies have demonstrated that the best mechanical performance of the cardiovascular system is found when the ventricle and the arterial tree are optimally matched (Sunagawa et al., 1983; Burkhoff and Sagawa, 1986; Elzinga and Westerhof, 1991). Accordingly, VA coupling has been considered as a central determinant of cardiovascular performance (Sunagawa et al., 1983; Starling, 1993).

Our data corroborate earlier reports about the compound nature of Ea_dyn_, being affected by both Ea and Ees, and then significantly related to VA coupling and LV_eff_. In light of these results, the definition of Ea_dyn_ as a dynamic assessment of arterial load (Monge Garcia et al., 2011, 2014, 2017a) could be understood in the same terms that the dynamic indexes of preload-responsiveness are related to cardiac preload. While all these indexes are not actual measures of preload, they are influenced by preload changes (Pinsky, 2012). Similarly, Ea_dyn_ cannot be considered as a true measure of afterload, although variations in afterload could affect to it. Therefore, afterload is to Ea_dyn_ as what the preload is to the index of preload-dependency.

Our results are in contrast with the recent report published by Bar et al. (2018). These authors retrospectively analyzed their previous results in 28 postoperative cardiac surgery patients suffering from vasoplegic syndrome, and they failed to demonstrate any relationship between Ea_dyn_ and VA coupling or its determinants (Guinot et al., 2018). One potential reason for this disagreement is the methodology used for determining Ees. In their study, Ees was estimated by the non-invasive single-beat method described by Chen et al. (2001), which is based on empirical estimation of a normalized population-averaged elastance and the data obtained from standard echocardiography and peripheral blood pressure measurements. Our results, however, were obtained from the well-validated method of the PV loops analysis from multiple-beat recordings during a transient preload reduction.

### Study Limitations

Our assessment of SVV was based on changes in LV volume obtained by the conductance technique, which requires a correction for the parallel conductance from the surrounding structures that can be determined and subtracted by the hypertonic saline method (Baan et al., 1984). Although changes in lung volume during ventilation could potentially affect the estimation of SV altering parallel conductance independently of the actual LV volume measurements, previous experimental studies have failed to show a systemic impact in parallel dual-field conductance volume signal during positive-pressure ventilation (Szwarc and Ball, 1998; Haney et al., 2006). Therefore, respiratory variation in SV during mechanical ventilation should reflect real SVV in our study. Second, there is a close physiological relationship between SVV and PPV, as the primary determinant of arterial pressure variations is the LV stroke volume changes induced by intermittent positive-pressure ventilation. Therefore, the close numbers could lead to a higher probability of random measurement error, mostly with lower values. Moreover, while PPV is directly obtained from the measurement of arterial pressure waveform, clinically SVV is often estimated using arterial pressure pulse-contour analysis or Doppler echocardiography. Therefore, the reliability of Ea_dyn_ will eventually rely on the accuracy of the SVV estimation and PPV measurements. Third, we focused only on the impact of different hemodynamic conditions on Ea_dyn_ during mechanical ventilation, but we did not evaluate how mechanical ventilation *per se* or respiratory mechanics could affect to the PPV/SVV relationship. Since we kept tidal volume and respiratory rate constant during each study and the lung and chest wall mechanics of each animal were normal, we suspect that the secondary impact of mechanical ventilation of cardiovascular state, independent of specific phasic changes in venous return induced by the small cyclic change in intrathoracic pressure, would be minimal. Finally, although our experimental protocol was designed to cover a broad range of hemodynamic conditions on an intact cardiovascular system, it may not reflect the complexity of a pathophysiological process, such as septic shock. Moreover, although we kept the level of anesthesia as low as possible during different experimental conditions, the study was performed in healthy female pigs submitted to anesthetic agents with known cardiovascular effects and potential influence over the visceral reflexes, so extrapolation of our results to human cardiovascular physiology should be done cautiously. Still, our data parallels the findings of several clinical studies. Furthermore, the influence of hyperoxia on the observed hemodynamic changes was unlikely, since the oxygen level, though not hypoxic, was not overly high to alter the cardiovascular system. Moreover, gender and age differences have been described in ventriculo-arterial coupling (Najjar et al., 2004). However, although we have no reference to analyze such differences, our 5.5 ± 0.8 months old female pigs were not subjected a long-term exposure to sex hormones (Andersson et al., 1999), which potentially may lead to lasting changes in the cardiovascular function of adult animals. Therefore, the influence of the pig gender in our study was likely negligible.

## Conclusion

Absolute Ea_dyn_ values and their change are defined by the combined effect of the LV contractility and LV afterload. Consequently, a significant relationship between Ea_dyn_, VA coupling and LV mechanical efficiency exists. Our results not only provide the physiological background for understanding Ea_dyn_ but also explain previous clinical results. Moreover, the concept of Ea_dyn_ as a potential easily measured bedside tool for monitoring VA coupling and LV_eff_ would allow to explore new applications of Ea_dyn_ as an index of cardiovascular performance and VA coupling.

## Data Availability Statement

The raw data supporting the conclusions of this article are available to any qualified researcher from the corresponding author upon reasonable request.

## Ethics Statement

The study was reviewed and approved by the Institutional Animal Care and Use Committee (IACUC) at the Edwards Research Center, and all procedures were performed in accordance with the USDA Animal Welfare Act regulations (AWArs), and the Guide for the Care and Use of Laboratory Animals (ILAR, NAP, Washington, DC, 2010, 8th edition).

## Author Contributions

MMG, MP, and MC contributed to the conception of the study. MMG, ZJ, and MP contributed to the design of the study. MMG, ZJ, and FH performed experimental research. MMG, ZJ, FH, JS, and MP analyzed and interpreted the data. All authors drafted the manuscript, reviewed it, contributed significantly to its critical review, approved the final version of the manuscript and ensure the accuracy or integrity of the results of this study and will be accountable for any question related to this work.

## Conflict of Interest

MMG was a consultant to Edwards Lifesciences and received honoraria and or travel expenses from Deltex Medical. MP was a consultant to Edwards Lifesciences, LiDCO Ltd., and Cheetah. MC has received honoraria and/or travel expenses from Edwards Lifesciences, LiDCO, Cheetah, Bmeye, Masimo, and Deltex Medical. ZJ, JS, and FH are Edwards Lifesciences employees.

## Footnotes

1 http://www.medcalc.org

## Abbreviations

C_art_: net arterial compliance
CI: confidence interval
CO: cardiac output
dP/dt_max_: Maximal ventricular pressure rise
E: elastance
Ea: effective arterial elastance
Ea_dyn_: dynamic arterial elastance
EDP: end-diastolic pressure
EDV: end-diastolic volume
Ees: end-systolic ventricular elastance
ESPVR: end-systolic pressure-volume relationship
ESV: end-systolic volume
IVC: inferior vena cava
LV: left ventricle
LVEF: left ventricular ejection fraction
LV_eff_: left ventricular mechanical efficiency
MAP: mean arterial pressure
P: pressure
PE: potential energy
Pes: end-systolic pressure
PPV: pulse pressure variation
PV: pressure-volume
PVA: pressure-volume area
SD: standard deviation
SV: stroke volume
SVV: stroke volume variation
SW: stroke work
TRP: total peripheral resistance
V: volume
V_0_: ventricular unstressed volume
VA: ventriculo-arterial.
